# Brachial-Ankle Pulse Wave Velocity as a Novel Modality for Detecting Early Diabetic Nephropathy in Type 2 Diabetes Patients

**DOI:** 10.1155/2021/8862573

**Published:** 2021-02-09

**Authors:** Xueyang Zhang, Ran Bai, Linxuan Zou, Junwei Zong, Yang Qin, Yongbo Wang

**Affiliations:** ^1^Department of Endocrinology, The First Affiliated Hospital of Dalian Medical University, Dalian, Liaoning, China; ^2^Department of Orthopedics, The First Affiliated Hospital of Dalian Medical University, Dalian, Liaoning, China

## Abstract

Brachial-ankle pulse wave velocity (baPWV) has been shown to correlate with a host of disorders associated with arterial stiffness. Type 2 diabetes is associated with the involvement of both small vessels and large vessels. Studies on the relevance of baPWV to early diabetic nephropathy are scarce. This retrospective observational case-control study enrolled 120 patients with type 2 diabetes from our medical records. We classified patients into two groups depending on the magnitude of albuminuria: 60 patients with microalbuminuria were classified as the early diabetic nephropathy group (EDN group) and 60 patients without albuminuria were classified as the diabetes without nephropathy group (DWN group). An additional 30 nondiabetic age- and sex-matched controls were also enrolled. Data regarding the lipid profile, blood pressure, baPWV, high-sensitivity C reactive protein (hs-CRP) level, anthropometric measurements, urine albumin/creatinine ratio (UACR), serum creatinine level, and glycemic control indices (i.e., fasting plasma glucose (FPG), postprandial glucose (PPG), and glycosylated hemoglobin (hemoglobin A1c, HbA1c)) were recorded for all enrolled participants. baPWV was significantly higher in the EDN group than in the DWN group. Moreover, baPWV was positively correlated with age, duration of diabetes, obesity, poor glycemic control, and high serum levels of triglycerides (TG), hs-CRP, creatinine, and uric acid as well as a high UACR (all *P* < 0.01). A significant negative correlation was found between baPWV and high-density lipoprotein levels (*P* < 0.05). Multivariate regression analysis showed that the hs-CRP level and duration of diabetes most strongly influenced baPWV. baPWV may be a convenient, noninvasive, and reproducible method for detecting early diabetic nephropathy.

## 1. Introduction

The prevalence of type 2 diabetes mellitus (T2DM) is rising globally, which is partly due to rapid urbanization and partly due to increased awareness and early diagnosis [1, 2]. The China Noncommunicable Disease Surveillance Group estimated that the prevalence in adults over 18 years of age was 9.7% in China at the end of 2010 [3]. In 2013, the overall prevalence of DM in the Chinese adult population was 10.4% [4]. The chronic complications of diabetes adversely affect the morbidity and mortality of diabetes patients while exacting a heavy toll on the socioeconomic status of individual patients and society at the same time [5, 6]. There has been a considerable increase in the prevalence of diabetic nephropathy (DN) due to the rising prevalence of diabetes, the earlier mean age at diagnosis of diabetes, and improved life expectancy of the general population due to medical advances [7]. DN is now one of the leading causes of end-stage renal disease requiring renal replacement therapies. Therefore, methods for the early detection of DN and for arresting its progression are receiving considerable medical attention globally.

According to various studies from China, 20%–40% of patients with type 2 diabetes have concomitant DN, and a significant portion of these have end-stage renal disease (ESRD) [8–11]. DN with ESRD in type 2 diabetes has a significantly higher mortality than diabetes without renal disease [12]. Early detection of DN, especially in early stages of the disease, is even more important, because it is preventable and even reversible if properly managed at that stage.

Pulse wave velocity (PWV) is the measurement of the speed of transit of the pressure wave along the arterial wall between two specified arterial end points. It is a good index of arterial stiffness; with higher stiffness, the velocity increases [13]. Brachial pulse wave velocity (baPWV) has been shown to be associated with various microangiopathic conditions, including hypertension, coronary heart disease, cerebrovascular diseases, and also complications of diabetes [14–16]. However, there have been very few studies on the role of baPWV measurement in the detection of DN. baPWV assessment by the oscillometric method is a quick, inexpensive, noninvasive, and reproducible method.

We undertook this case-control observational study to elucidate the utility of baPWV measurement in patients with early DN (EDN) diagnosed on the basis of microalbuminuria detected as an elevated urine albumin/creatinine ratio (UACR). We also performed correlation and regression analyses of baPWV with other confounding risk factors for arterial stiffness.

## 2. Materials and Methods

### 2.1. Participants

From our medical records, we selected 120 patients with type 2 diabetes (68 males, 52 females), diagnosed according to the World Health Organization criteria published in 2018, who had visited the First Affiliated Hospital of Dalian Medical University between July 2014 and January 2015. The study protocol was in accordance with the Declaration of Helsinki and was approved by the ethics committee of the First Affiliated Hospital of Dalian Medical University. Written informed consent was obtained from all participants after enrollment in the study. Thirty nondiabetic control participants were also recruited.

Patients between 35 and 70 years of age were included in the study. Patients with a duration of diabetes exceeding 15 years and patients with a past history or present evidence of coronary heart disease, cerebrovascular disease, or peripheral arterial disease were excluded from the study. Patients with type 1 diabetes or other forms of diabetes were excluded. Patients with acute complications of diabetes, such as diabetic ketoacidosis, hyperglycemic hyperosmolar status, lactic acidosis, and hypoglycemic coma within the previous 6 months were also excluded from the study. Patients with severe renal failure (estimated glomerular filtration rate (eGFR) < 60 mL/min/1.73 cm^2^) or hepatic dysfunction (serum alanine aminotransferase > 80 U/L) were also excluded from enrollment. According to the UACR estimation, patients were divided into two groups: the EDN group (UACR 30–300 *μ*g/mg) and the DWN group (UACR < 30 *μ*g/mg).

### 2.2. Laboratory Parameters

Anthropometric data including height, weight, waist circumference, and body mass index (BMI) were recorded. Systolic blood pressure and diastolic blood pressure were recorded to calculate pulse pressure (PP) and mean arterial pressure (MAP). Glycemic control was assessed by measuring fasting plasma glucose (FPG), postprandial glucose (G-6-PDH method), and HbA1c. Lipid profile (direct method), ALT (International Federation of Clinical Chemistry method), AST (IFCC method), uric acid (urase method), and creatinine (HMMPS (N-(3-sulfopropyl)-3-methoxy-5-methylaniline) method) estimation were done using routine laboratory techniques. Serum fasting C peptide (FCP) and 2-hour postprandial C peptide (2hCP) were examined by electrochemiluminescence assay (LIAISON XL, Germany). Levels of high-sensitivity C-reactive protein (hs-CRP) were measured by the immunoturbidimetric method (SIEMENS BN, Germany). HbA1c was determined by high-performance liquid chromatography (Premier Hb9210, China).

### 2.3. Measurement of UACR

Second-pass morning urine specimens were collected for 3 consecutive days. Urinary albumin excretion was measured by the immunoturbidimetric method, and urinary creatinine was determined by an enzymatic method. These parameters were used to calculate the UACR [17, 18]. The mean of three readings was used in the present study.

### 2.4. Measurement of baPWV

baPWV was calculated by dividing the distance (in centimeters) between two arterial end points (brachial and ankle) by the transit time (in seconds) the pressure waveform took to travel. The distance between the peripheral arterial end sites was calculated using a height-based algorithm, as required by the Colin waveform analyzer [19]. Transit time was calculated using the foot-to-foot velocity method [20]. The average of two runs was used for analysis.

### 2.5. Statistical Analysis

The normality of continuous variables was verified by the Kolmogorov-Smirnov test. Normally distributed variables were expressed as mean ± standard deviation (SD), and differences among the three groups were analyzed by one-way analysis of variance (ANOVA). The nonnormally distributed variables were expressed as median ± interquartile range (IQR), and differences in such variables among the three groups were identified by the nonparametric Kruskal-Wallis test. The Levene test was used to verify the homogeneity of variance. Correlation between baPWV and continuous variables was analyzed by Pearson linear correlation (for normally distributed data) or Spearman rank correlation (for skewed data). Multiple regression analysis was performed to identify variables that had an independent effect on baPWV. Receiver operating characteristic (ROC) curve analysis was done to identify a specific cut off with the help of Youden's index that gave the best specificity and sensitivity for detection of diabetic nephropathy defined by the presence of albuminuria. SPSS 25.0 software was used for all statistical analyses, and the level of statistical significance was set at *P* < 0.05.

## 3. Results

### 3.1. Comparison of Baseline Clinical and Laboratory Data among the Three Groups

**Figure 1:**
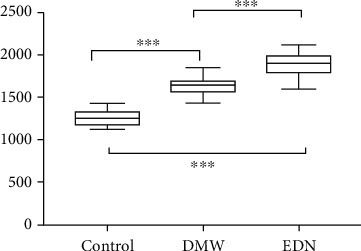
Box plot of baPWV measurements in the three groups. baPWV was significantly higher in patients with diabetes (with or without DN) than in the control group. Patients with DN had a significantly higher baPWV than diabetes patients without nephropathy. ^∗∗∗^*P* < 0.001.

The baseline clinical and laboratory data are presented in Table 1. Patients with diabetes were significantly more obese, as seen by a higher BMI, than the control population. Patients with diabetes (both with and without DN) had significantly higher low-density lipoprotein cholesterol (LDL-C) levels, significantly lower high-density lipoprotein cholesterol (HDL-C) levels, significantly higher blood pressure, and poorer glycemic control parameters as compared to the control population, as expected. There were no significant differences in age, waist circumference, total cholesterol, and TG levels between the three groups. Patients in the EDN group had a significantly longer duration of diabetes than patients in the DWN group. Patients in the EDN group also had poorer glycemic control, as evidenced by a significantly higher HbA1c, as compared to those in the DWN group. Patients in the EDN group had significantly higher hs-CRP and serum creatinine levels and significantly lower eGFR levels as compared to those in the DWN group. Moreover, baPWV was significantly higher in patients with diabetes (EDN and DWN groups) than in the control participants. Finally, patients with EDN had a significantly higher baPWV than those in the DWN group (Table 1 and Figure 1).

### 3.2. ROC Curve Analysis of baPWV

**Figure 2:**
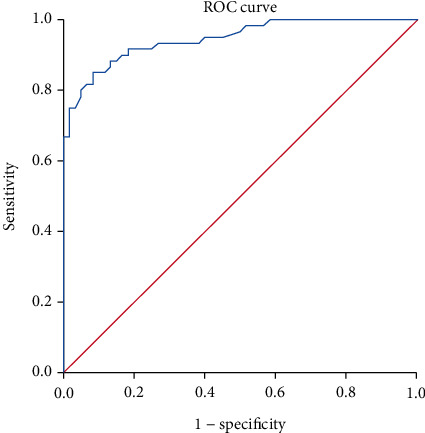
ROC curve for the ability of baPWV to detect DN. The area under the ROC curve was 0.946, with a 95% CI of 0.908–0.983, indicating that baPWV measurement is an effective method for the detection of EDN. The optimal cut-off value of baPWV was 1716 cm/s, with a sensitivity of 91.7% and a specificity of 81.7%.

ROC curve analysis was performed to evaluate the effectiveness of baPWV for detecting DN (Figure 2). The area under the ROC curve (AUC) was 0.946, with a 95% confidence interval (CI) of 0.908 to 0.983, indicating that baPWV is an effective method for detecting EDN. The optimal cut-off value for baPWV was 1716 cm/s, with a sensitivity of 91.7% and a specificity of 81.7%.

### 3.3. Correlation between baPWV and Other Variables

Correlation analysis showed that baPWV was correlated significantly with age and duration of diabetes. baPWV also showed positive correlations with anthropometric parameters such as BMI and waist circumference. Mean arterial blood pressure, HbA1c, FPG, and postprandial plasma glucose values also were significantly positively correlated with baPWV. Moreover, baPWV correlated positively with hs-CRP, serum creatinine, microalbuminuria, and TG levels. Conversely, HDL-C showed a significant negative correlation with baPWV (Table 2).

### 3.4. Multiple Linear Regression Analysis of Risk Factors for Elevated baPWV

Multiple regression analysis (Table 3) showed that the duration of diabetes, hs-CRP level, FPG, and BMI were independent risk factors for elevated baPWV, while the HDL-C level was an independent protective factor for baPWV. Duration of diabetes and hs-CRP level emerged as the strongest predictive variables for elevated baPWV.

## 4. Discussion

DN is an insidious, gradually progressive chronic complication of diabetes, which has a multifactorial pathogenesis influenced by glycemic control, blood pressure control, lipid parameters, genetic factors, and environmental and inflammatory factors. Internationally, microalbuminuria detection by UACR determination has been accepted as the gold standard for early detection of DN [21]. Spot sample UACR is a sensitive, reliable, inexpensive, and convenient method for detecting and quantifying albumin excretion, as opposed to 24-hour urine collection, which is a cumbersome procedure.

UACR however has certain limitations as well; for example, conditions such as accelerated blood pressure, heart failure, fever, urine infection, and systemic illness may result in falsely positive results. To minimize these confounding variables, two positive readings 3 months apart are needed to diagnose DN [22]. This leads to some delay in diagnosis as well. Also, certain patients with DN do not show increased albuminuria and have the so-called nonalbuminuric variety of DN. Hence, new modalities are needed for the diagnosis of DN that overcome the limitations of UACR estimation.

Measurement of baPWV is a new, noninvasive, and reproducible method for assessing arterial stiffness [23]. Previous studies have demonstrated that traditional risk factors associated with atherosclerosis, such as age, diabetes mellitus, dyslipidemia, obesity, and hypertension, influence baPWV. The baPWV of normal healthy persons is less than 1400 cm/s [24]. In the present study, the baPWV of diabetes patients was higher than that of the healthy controls (1253.8 ± 79.35 cm/s), and the baPWV of diabetes patients with EDN (1889.2 ± 126.72 cm/s) was also higher than that of diabetes patients without nephropathy (1626.9 ± 95.99 cm/s). For a baPWV cut-off value of 1716 cm/s, the sensitivity was 91.7% and the specificity was 81.7% for the detection of DN. As the process of atherosclerosis proceeds, fibrosis in the vessel wall ensues, which leads to increased stiffness and decreased compliance, further leading to increased velocity of waveform transmission and hence a higher pulse wave velocity [25].

Smulyan et al. claimed that age and blood pressure were the strongest independent determinants of baPWV [26]. Yiming et al. found that baPWV was relatively strongly associated with age and blood pressure and weakly associated with BMI, TG level, and glycemic status [27]. In the present study, baPWV was positively correlated with age, duration of diabetes, BMI, waist circumference, MAP, FPG, 2hPPG, HbA1c, TG, hs-CRP, and UACR and negatively correlated with HDL-C. These findings are similar to the results of Gomez-Sanchez et al., who found that metabolic syndrome and most of its individual components are associated with an elevated baPWV [28]. baPWV was highest when the metabolic syndrome components of increased blood pressure, FPG, and waist circumference were concurrent. baPWV increases with age due to degeneration and decreased elasticity of the arterial wall. In the study by Zhao et al., all lipid profile parameters were correlated with arterial stiffness as assessed by baPWV, and HDL-C had a protective effect on arterial stiffness [29].

Increased arterial stiffness plays a key role in the progression of chronic kidney disease [30]. A series of studies reported a linear association between renal impairment and arterial stiffness [31–33]. Arterial stiffness is therefore the link between baPWV and DN. Proinflammatory cytokines play an important role in the establishment of arteriosclerosis and consequently in chronic kidney injury [34, 35]. hs-CRP is one of the acute phase reaction proteins, and hs-CRP levels reflect the degree of chronic low-grade systemic inflammatory response. A previous study demonstrated that hs-CRP levels are elevated in patients with DN [36].

The present study has several limitations. First, the sample size was small, and hence, further multicenter clinical trials are needed to collect robust data across different ethnic populations to assist in clinical decision making. Second, other arterial stiffness indices, such as carotid-femoral PWV and central pulse wave analysis, were not investigated in this study [20]. More studies evaluating these other arterial stiffness indices are needed to corroborate our findings. Follow-up studies are also needed to determine whether patients with high baPWV but no albuminuria will later develop albuminuria. Finally, studies are needed to investigate whether drugs like statins, antiplatelet agents, and vasodilators have any influence on baPWV measurements.

In summary, baPWV measurement has the potential for wide-scale application for detection of EDN, and further studies are warranted to establish its role in predicting future risk of DN.

## Figures and Tables

**Table 1:** Baseline clinical, diabetic, blood lipid, inflammation, and renal function parameters in the control, DWN, and EDN groups.

	Control (*n* = 30)	DWN (*n* = 60)	EDN (*n* = 60)	*P*	*P*′	*P* ^″^	*P* ^‴^
baPWV^△^ (cm/s)	1253.8 ± 79.35	1626.9 ± 95.99	1889.2 ± 126.72	<.001	<.001	<.001	<.001
Age^△^ (y)	59.6 ± 5.5	59.1 ± 6.4	61.4 ± 5.6	.078			
Gender (M/F)	17/13	27/33	26/34	.463			
Duration of diabetes^#^ (y)	0	6.25 ± 4.25	10 ± 8	<.001	<.001	<.001	<.001
BMI^△^ (kg/m^2^)	23.7 ± 0.98	25.5 ± 1.78	26.1 ± 1.71	<.001	<.001	<.001	.041
WC^#^ (cm)	83.5 ± 79.88	85 ± 81	85 ± 81	.139			
MAP^△^ (mmHg)	93.4 ± 6.93	93.2 ± 6.64	101.5 ± 5.98	<.001	.896	<.001	<.001
PP^#^ (mmHg)	40 ± 38.75	52.5 ± 50	55 ± 50	<.001	<.001	<.001	.518
FPG^△^ (mmol/L)	5.1 ± 0.57	8.0 ± 1.22	9.1 ± 1.33	<.001	<.001	<.001	<.001
2hPPG^△^ (mmol/L)	7.1 ± 0.53	13.4 ± 2.49	13.9 ± 2.81	<.001	<.001	<.001	.318
FCP^#^ (ng/mL)	2.4 ± 2.05	1.86 ± 1.29	1.86 ± 1.15	.001	.003	.001	1
2hCP^△^ (ng/mL)	7.6 ± 1.49	7.1 ± 2.88	6.4 ± 1.61	.034	.273	.013	.085
HbA1c^#^ (%)	4.75 ± 4.38	6.2 ± 5.65	7.1 ± 6.5	<.001	<.001	<.001	<.001
TC^#^ (mmol/L)	4.7 ± 4.37	4.77 ± 4.6	4.86 ± 4.7	.482			
TG^#^ (mmol/L)	1.56 ± 1.16	1.77 ± 1.33	1.86 ± 1.37	.052			
HDL-C^#^ (mmol/L)	1.27 ± 1.06	1.18 ± 1.09	1.1 ± 0.96	.040	1	.058	.190
LDL-C^#^ (mmol/L)	2.24 ± 1.82	2.67 ± 2.36	2.68 ± 2.38	.018	.040	.022	1
hs-CRP^△^ (mg/L)	0.7 ± 0.15	2.2 ± 0.38	5.4 ± 1.06	<.001	<.001	<.001	<.001
Creatinine^△^ (*μ*mol/L)	71.4 ± 6.71	78.1 ± 13.11	92.5 ± 11.85	<.001	.011	<.001	<.001
Urea^△^ (*μ*mol/L)	5.7 ± 1.16	6.1 ± 0.71	6.7 ± 1.00	<.001	.052	<.001	<.001
UA^#^ (*μ*mol/L)	324 ± 297.5	325.5 ± 303.5	358 ± 310	.004	1	.011	.028
UACR^#^ (*μ*g/mg)	6.25 ± 3.58	25.5 ± 20.83	256.7 ± 228.78	<.001	<.001	<.001	<.001
eGFR^△^ (mL/min)	99.3 ± 10.22	93.0 ± 14.98	73.7 ± 12.89	<.001	<.001	<.001	<.001

baPWV = brachial-ankle pulse wave velocity; BMI = body mass index = body weight (kg)/height^2^(m); WC = waist circumference; MAP = mean arterial pressure; PP = pulse pressure; FPG = fasting venous plasma glucose; 2hPPG =2-hour postprandial venous plasma glucose; FCP = fasting C peptide; 2hCP =2-hour postprandial C peptide; HbA1c = hemoglobin A1c; TC = total cholesterol; TG = total triglycerides; HDL-C = high-density lipoprotein cholesterol; LDL-C = low-density lipoprotein cholesterol; hs-CRP = high-sensitivity C reactive protein; UA = uric acid; UACR = urine albumin/creatinine ratio. ^△^One-way ANOVA was carried out, if the variable followed a normal distribution by the Kolmogorov-Smirnov test. ^#^Nonparametric Kruskal-Wallis test was carried out, if the variable did not follow a normal distribution by the Kolmogorov-Smirnov test. *P* for all three groups; *P*′ for the control group vs. the DWN group; *P*^″^ for the control group vs. the EDN group; *P*^‴^ for the DWN group vs. the EDN group.

**Table 2:** Correlation analysis of baPWV and other variables.

	*R*	*P*
Age^△^ (y)	0.216	.008
Duration of diabetes^#^ (y)	0.875^∗^	<.001
BMI^△^ (kg/m^2^)	0.416^∗^	<.001
WC^#^ (cm)	0.167	.041
MAP^△^ (mmHg)	0.376^∗^	<.001
PP^#^ (mmHg)	0.446^∗^	<.001
FPG^△^ (mmol/L)	0.729^∗^	<.001
2hPPG^△^ (mmol/L)	0.601^∗^	<.001
FCP^#^ (ng/mL)	-0.13	.113
2hCP^△^ (ng/mL)	-0.125	.128
HbA1c^#^ (%)	0.586^∗^	<.001
TC^#^ (mmol/L)	0.098	.233
TG^#^ (mmol/L)	0.178	.03
HDL-C^#^ (mmol/L)	-0.181	.027
LDL-C^#^ (mmol/L)	0.129	.117
hs-CRP^△^ (mg/L)	0.816^∗^	<.001
Creatinine^△^ (*μ*mol/L)	0.467^∗^	<.001
Urea^△^ (*μ*mol/L)	0.364^∗^	<.001
UA^#^ (*μ*mol/L)	0.233	.004
UACR^#^ (*μ*g/mg)	0.827^∗^	<.001

baPWV = brachial-ankle pulse wave velocity; BMI = body mass index = body weight (kg)/height^2^(m); WC = waist circumference; MAP = mean arterial pressure; PP = pulse pressure difference; FPG = fasting venous plasma glucose; 2hPPG =2-hour postprandial venous plasma glucose; FCP = fasting C peptide; 2hCP =2-hour postprandial C peptide; HbA1c = hemoglobin A1c; TC = total cholesterol; TG = total triglycerides; HDL-C = high-density lipoprotein cholesterol; LDL-C = low-density lipoprotein cholesterol; hs-CRP = high-sensitivity C reactive protein; UA = uric acid; UACR = urine albumin/creatinine ratio. ^△^Pearson linear correlation analysis was carried out, if the variable followed a normal distribution by the Kolmogorov-Smirnov test. ^#^Spearman rank correlation analysis was carried out, if the variable did not follow a normal distribution by the Kolmogorov-Smirnov test. ^∗^*P* < 0.001.

**Table 3:** Multivariate regression analysis for factors influencing baPWV.

	Unstandardized		Standardized *β*	*t*	*P*	Collinearity	
	*β*	SD				Tolerance	VIF
Constant	1015.25	115.345		8.802	<.001		
Duration of diabetes (y)	33.324	2.893	0.583	11.521	<.001	0.337	2.968
hs-CRP (mg/L)	37.878	5.678	0.299	6.671	<.001	0.429	2.332
FPG (mmol/L)	15.961	6.046	0.115	2.64	.009	0.453	2.206
BMI (kg/m^2^)	9.859	4.527	0.071	2.178	.031	0.818	1.223
HDL-C (mmol/L)	-31.164	14.438	-0.065	-2.158	.033	0.964	1.037

baPWV = brachial-ankle pulse wave velocity; hs-CRP = high-sensitivity C reactive protein; FPG = fasting venous plasma glucose; BMI = body mass index = body weight (kg)/height^2^(m); HDL-C = high-density lipoprotein cholesterol.

## Data Availability

The clinical data used to support the findings of this study are available from the corresponding author upon request.
